# Spatio-temporal patterns of malaria infection in Bhutan: a country embarking on malaria elimination

**DOI:** 10.1186/1475-2875-10-89

**Published:** 2011-04-16

**Authors:** Kinley Wangdi, Jaranit Kaewkungwal, Pratap Singhasivanon, Tassanee Silawan, Saranath Lawpoolsri, Nicholas J White

**Affiliations:** 1 Department of Tropical Hygiene, Faculty of Tropical Medicine, Mahidol University, Bangkok, Thailand; 2 Phuentsholing General Hospital, Phuentsholing, Bhutan; 3 The Rural Health Training and Research Centre, Faculty of Public Health, Mahidol University, Bangkok, Thailand; 4 Mahidol-Oxford Unit, Faculty of Tropical Medicine, Mahidol University, Bangkok, Thailand

## Abstract

**Background:**

At the verge of elimination of malaria in Bhutan, this study was carried out to analyse the trend of malaria in the endemic districts of Bhutan and to identify malaria clusters at the sub-districts. The findings would aid in implementing the control activities. Poisson regression was performed to study the trend of malaria incidences at district level from 1994 to 2008. Spatial Empirical Bayesian smoothing was deployed to identify clusters of malaria at the sub-district level from 2004 to 2008.

**Results:**

Trend of the overall districts and most of the endemic districts have decreased except Pemagatshel, which has an increase in the trend. Spatial cluster-outlier analysis showed that malaria clusters were mostly concentrated in the central and eastern Bhutan in three districts of Dagana, Samdrup Jongkhar and Sarpang. The disease clusters were reported throughout the year. Clusters extended to the non-transmission areas in the eastern Bhutan.

**Conclusions:**

There is significant decrease in the trend of malaria with the elimination at the sight. The decrease in the trend can be attributed to the success of the control and preventive measures. In order to realize the target of elimination of malaria, the control measure needs to be prioritized in these high-risk clusters of malaria.

## Background

Since the first malaria survey of Bhutan in 1962, malaria cases decreased gradually over the years. The first case of malaria was reported in 1965, there were 518 cases. The highest number of cases was recorded in 1994 with 39,852 cases and the highest death was recorded in 1994 with 62 deaths [1]. The Annual Parasite Incidence (API) per 1,000 population at risk of malaria in Bhutan was 3.98% in the year 2006 [2]. Bhutan contributed second lowest number of cases after Sri Lanka with 1,868 cases in SEARO region in the same year [2].

The control measures have also changed over the years. Indoor residual spraying (IRS) using dichlorodiphenyltrichloroethane (DDT) was implemented since 1964 with three rounds and later two rounds until 1994. With the reports of resistance to DDT in some parts of world, DDT was replaced by deltamethrin (synthetic pyrethoid) from 1994 till 1997 [3,4]. Insecticide treated-bed nets (ITN) became the main control strategy from 1998 with focal IRS during outbreaks and emergencies, and high *Plasmodium falciparum *transmission areas with API >10%. Long-lasting insecticide-treated nets (LLINs) have proven to reduce the mortality and morbidity of malaria by reducing the man-mosquito contact [5], so Bhutan distributed over 100,000 LLINs with the grants from Global Fund to Fight against AIDS, Tuberculosis and Malaria (GFATM), as of 2006 [1]. The diagnosis and treatment of malaria is integrated with the general health care system with early diagnosis and prompt treatment remaining the cornerstone of the malaria control. Microscopic facilities for early diagnosis are extended to all the health centres in these endemic districts. Microscopy is further supplemented with rapid diagnostic kits in most of the health facilities. Uncomplicated *P. falciparum *is treated with the combination of artemether and lumefantrine (Coartem^®^) with different regimen for the pregnant woman. The present regimen was initiated since 2006. While the treatment for *Plasmodium vivax *has not changed and is treated with chloroquine for three days and primaquine administered over 14-days [6,7].

Disease surveillance connotes the ongoing systematic collection, analysis, and interpretation of out-come specific data for use in planning, implementation, and evaluation of public health practice [8]. Its purpose is to detect changes in trends or distribution, so that necessary control and preventive measures can be initiated. Malaria surveillance includes laboratory confirmation of presumptive diagnosis, finding out the source of infection and identification of all cases and susceptible contacts and still others who are at risk, in order to prevent further spread of the disease. The ultimate objective of malaria surveillance is prevention and control of malaria in the community [9].

In Bhutan, malaria surveillance is a vertical system and through the passive reporting of the fever cases and the microscopically confirmed malaria cases. Therefore, every health centre; basic health unit II (BHU II), basic health unit I (BHU I), district hospital, regional and national referral hospital reports the malaria cases to Vector-borne Disease Control Programme (VDCP). Every week, the health centres in the endemic districts report the number of fever cases to the programme. At the end of every month, microscopically confirmed malaria cases are reported from the hospitals and health centres to VDCP. The fever surveillance serves as the early warning system so that prompt and early investigation can be initiated. In case of outbreaks, necessary interventions and control measures can be implemented.

As per the WHO guidelines, 10% of negative slides and 25% of positive cases are sent to the VDCP for quality control. As a national health policy, every fever patient that reports to health centres in the endemic districts and in the other non-endemic districts with a history of travel to malaria endemic areas in the past two weeks are needed to be tested for malaria either with the microscopy or rapid diagnostic test (RDT). However, the positive cases with RDT are further confirmed microscopically.

At the programme level, the reported fever and malaria cases are analysed such as the recent trends and information is fed back to the health centres. In case of unusually large numbers of fever and malaria cases, the health centres which report the unusual episodes are facilitated to carry out the investigations for possible causes of unusual fever or malaria cases for early containment and control.

## Methods

### Study districts

**Figure 1:**
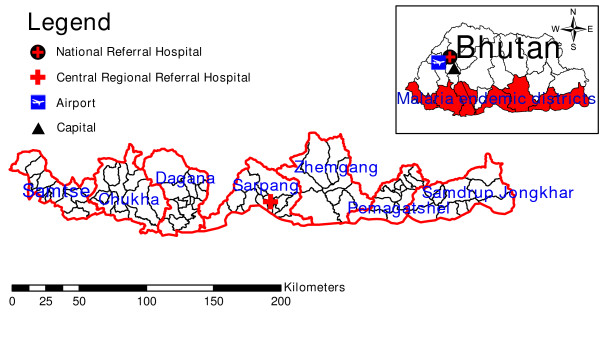
**Map showing the study area**.

The study districts lie in the southern part of Bhutan neighbouring Indian states of Assam and West Bengal. A total of seven malaria endemic districts were included in the study. These districts were further divided into 84 sub-districts (Figure 1). The total population under the study as of 2008 was 277,257. People living in these malaria endemic districts are expected to be at risk for malaria.

The health services to the sub-districts are provided through outreach clinic (ORC) and BHU II. The districts also have district hospitals with facilities for investigation such as laboratory and x-ray services. These district hospitals also serve as the referral points for the BHUs under the same district. There are regional referral hospitals, which are tertiary centres for the districts with specialist services. The central regional referral hospital is located in one of the district under the study. There are a total of 13 hospitals, eight BHU I, 62 BHU II and 199 ORCs (Table 1) [10]. These various health facilities and services are likely to serve almost all population in the area.

**Table 1:** Health facilities under the study in endemic districts.

Districts	Types of health centres			
	
	Hospital	BHU I	BHU II	ORC
**Chukha**	**3**	**1**	**8**	**47**
**Dagana**	**1**	**2**	**6**	**16**
**Pemagatshel**	**1**	**1**	**11**	**33**
**Samdrup Jongkhar**	**2**	**2**	**6**	**36**
**Samtse**	**3**	**0**	**9**	**18**
**Sarpang**	**2**	**0**	**10**	**11**
**Zhemgang**	**1**	**2**	**12**	**38**

**Total**	**13**	**8**	**62**	**199**

Source: Annual Health Bulletin 2010.

### Overall malaria occurrence

The data for the study was extracted from the database of VDCP programme, Department of Public Health, Ministry of Health. All microscopically-confirmed malaria cases observed in any BHU IIs, BHU Is and hospitals are regularly reported to the VDCP. In this study, monthly malaria occurrences during 1994 to 2008 at the district level and 2004 to 2008 at the sub-district level were obtained from the database for the analysis.

### Trend analysis

Trend analysis in this study was assessed using Poisson regression. Poisson regression is a type of a Generalised Linear Model (GLM) model that follows a Poisson distribution. This regression model is used to model discrete events that occur infrequently in time or space; hence, it is sometimes called the distribution of rare events [11]. In this study, the distribution of microscopically confirmed malaria cases (events) occurred under baseline population across years and districts were assumed to follow the Poisson distribution. Then the Poisson regression model was performed to calculate the incidence rate ratio and 95% confidence interval was calculated using the Poisson regression model to determine the change in malaria incidence over years, stratified by districts.

### Malaria clusters

The sub-districts analysis of the malaria in the endemic districts and mapping of the malaria clusters was done using the five-year malaria surveillance data from 2004 to 2008. Spatial Empirical Bayesian (SEB) was used to minimize the phenomenon of the Modifiable Areal Unit Problem (MAUP). The clusters were observed at the sub-district level at the monthly period over five years.

The Moran's *I *statistics was used to calculate the autocorrelation or global clusters of malaria incidence rates of different sub-districts.

The Moran's I coefficient can be defined as;

Where *W*_*ij *_= is a measure of the closeness of areas *i *and *j*; *W*_*ij*_= 1 if areas *i *and *j *are adjacent (have a common boundary) and 0 otherwise

*Z*_*i *_= could be the residuals (*O*_*i *_- *E*_*i*_) or standard mortality or morbidity ratio (SMR) of an area/attribute value for area *i*

*Z*_*j *_= attribute value for area *j*

 = mean value in study region

*n *= the numbers of areas

*I *= Moran's I

The spatial weights indicate whether regions neighbour each other. Spatial weights based on contiguity matrix in this research are queen contiguity.

The significance of Moran's I was assessed using 999 Monte Carlo randomization. The p-value of 0.05 or less was considered as significant [12,13]. LISA refers to a spatial autocorrelation at the local scale or local clusters. LISA statistic was calculated as:

Where *Z*_*i *_and Z _*j *_are the deviations from the mean for the corresponding X values, or

*δ *= standard deviation of the variable X

*Z*_*i *_= Z-score of *X*_*i*_

*W*_*ij *_= spatial weights matrix on row-standardized form

LISA detects clusters of either similar or dissimilar disease frequency values around observations. The sum of the LISA for all observations is proportional to the global Moran's I statistic [12,14].

## Results

### Malaria occurrence

Malaria in Bhutan is mostly reported by seven districts namely: Chukha, Dagana, Pemagatshel, Samdrup Jongkhar, Samtse, Sarpang and Zhemgang (Table 2). The highest malaria cases were reported in the age group of 15-45 years with male getting infection more than female. This is followed by age group of 5-14 years; the infants were infected with malaria the least followed by 1-4 years (Table 3).

**Table 2:** Malaria cases of the endemic districts of Bhutan from 2004 to 2008.

Districts	2004		2005		2006		2007		2008	
	
	Cases	%	Cases	%	Cases	%	Cases	%	Cases	%
**Chukha**	191	7.2	167	9.2	199	10.7	55	6.9	25	7.6
**Dagana**	4	0.2	2	0.1	8	0.4	3	0.4	11	3.3
**Pemagatshel**	5	0.2	4	0.2	11	0.6	3	0.4	12	3.67
**Samdrup Jongkhar**	374	14.0	506	27.7	618	33.1	306	38.6	86	26.1
**Samtse**	1018	38.1	344	18.9	248	13.3	59	7.4	17	5.2
**Sarpang**	910	34.1	656	36.0	570	30.5	286	36.1	136	41.3
**Zhemgang**	42	1.6	33	1.8	39	2.1	3	0.4	4	1.2
**Other districts**	126	4.7	113	6.2	175	9.4	78	9.8	38	11.6

**Total**	2670	100	1825	100	1868	100	793	100	329	100

**Table 3:** Different age break down of the malaria cases from 2004 to 2007.

Age	2004			2005			2006			2007		
	
	M	F	T	M	F	T	M	F	T	M	F	T
**<1**	12	0	**12**	5	2	**7**	9	3	**12**	3	1	**4**
**1-4**	85	66	**151**	55	55	**110**	55	47	**102**	17	10	**27**
**5-14**	357	292	**649**	197	177	**374**	212	152	**364**	82	69	**151**
**15-49**	1000	552	**1552**	743	371	**1114**	848	350	**1198**	359	156	**515**
**>50**	192	114	**306**	136	84	**220**	132	60	**192**	67	39	**96**

**Total**	**1646**	**1024**	**2670**	**1136**	**689**	**1825**	**1256**	**612**	**1868**	**518**	**275**	**793**

Note: M - male, F - female and T - total.

### Trend analysis

The trend analysis of the overall endemic districts showed a statically significant decrease in the trend (IRR = 0.94 ± 0.0001) over the study period from 1994 to 2008 (p < 0.001). The six endemic districts showed decreased trend over the study period; Chukha district with (IRR = 0.97 ± 0.0003), Dagana district (IRR = 0.98 ± 0.001), Samdrup Jongkhar (IRR = 0.96 ± 0.0001), Samtse district (IRR = 0.98 ± 0.0002), Sarpang (IRR = 0.97 ± 0.0001) and Zhemgang (IRR = 0.99 ± 0.0005), except Pemagatshel district, which saw an increased trend with IRR = 1.02 ± 0.0025 (Table 4).

**Table 4:** Incidence rate ratio, standard error and 95% confidence interval (CI) of seven endemic districts and the overall endemic districts.

Districts	IRR	SE	95% CI
Chukha	0.97	0.0003	0.97-0.97
Dagana	0.98	0.001	0.98-0.98
Pemagatshel	1.02	0.0025	1.01-1.02
Samdrup Jongkhar	0.96	0.0001	0.97-0.98
Samtse	0.98	0.0002	0.98-0.98
Sarpang	0.97	0.0001	0.97-0.97
Zhemgang	0.99	0.0005	0.98-0.98

All endemic districts	0.94	0.0001	0.94-0.94

IRR - incidence rate ratio; SE - standard error; CI - confidence interval

### Malaria clusters

**Figure 2:**
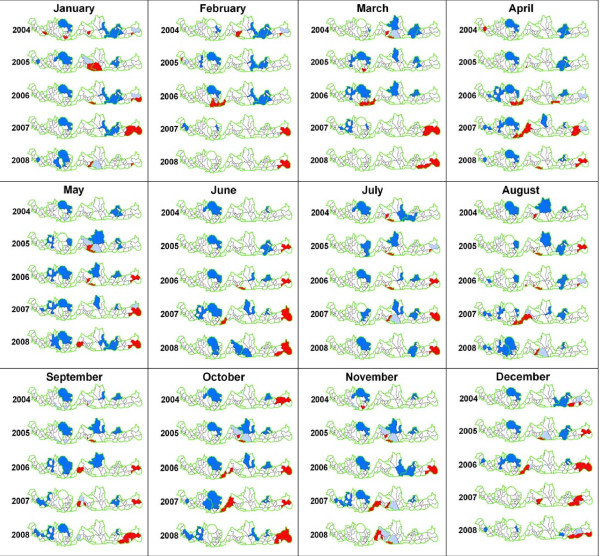
**Malaria clusters of the sub-districts of the endemic districts of Bhutan from 2004 to 2008**. Colour codes: Red = high-high, Dark blue = low-low, Light blue = low-high, Pink = high-low

Mapping of malaria clusters at the sub-districts of the seven endemic districts showed that the clusters of malaria were confined to the central and eastern part of Bhutan (Figure 2). For the month of January, clusters were reported from different sub-districts of the country for 2004, but malaria clusters started to be confided to the central and eastern part for the rest of the study period. In February and March, clusters for 2004 and 2006 occurred in the central part while malaria clusters for 2007 and 2008 occurred in the eastern sub-districts. From 2006 to 2008, clusters were located at the central and eastern Bhutan for April month. In May and June, malaria clusters became more pronounced in eastern Bhutan. Malaria clusters for July remained same as the previous month and extended to the central region. The clusters for August of the eastern region decreased while in the central region increased over the five years. In September, malaria clusters returned to the eastern sub-districts with further increase to the neighbouring sub-districts and into the central region for the rest of the months (Figure 2).

## Discussion

### Overall malaria occurrence

Malaria in Bhutan is mostly reported by seven districts namely: Chukha, Dagana, Pemagatshel, Samdrup Jongkhar, Samtse, Sarpang and Zhemgang (Table 2 and Figure 1). Most of the transmission usually takes place in the summer months when ambient temperature is very favourable. These districts have sub-tropical climate with hot summers, temperatures reaching as high as 30°Celsius; most of the districts have abundant rainfall with humidity above 60% throughout the year [15]. The transmission within the districts vary greatly for a number of reasons, such as varying in altitude, climatic factors and environmental factors of sub-districts that make up districts.

**Figure 3:**
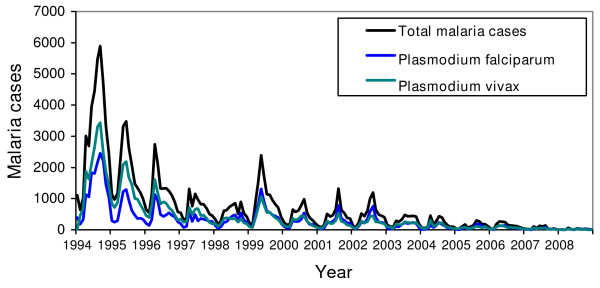
**Total malaria cases (black line), *P. falciparum *(blue line) *P. vivax *(red line) of the endemic districts of Bhutan from 2004 to 2008**.

The overall occurrence of malaria decreased during the study period throughout the study area (Figure 3). This can be attributed to a number of reasons, such as the enhanced control measures, or change in the treatment modules. Most malaria infection occurred in the age group of 15-45 years among males, which is suggestive of the transmission not being indigenous rather imported malaria. However, this could be related to the differences in health service utilization or differences in behavioral exposure to disease. Nevertheless, further studies need to be carried out to explore the behavioral factors associated to the disease in this population.

### Trend analysis

There appears to have a seasonal pattern with two peaks, first smaller peak just before the onset of the summer and the bigger peak during the summer (June-August) when rainy season starts. The small peak could be due to the increased in temperature just before the summer, which enhance the vector multiplication and development. Majority of rural population of Bhutan depends on agriculture for their livelihood. Therefore, people would be exposed to the mosquito bite during this time while they are involved in agricultural activities. The second bigger peak would be associated with the summer months, where rainfall is abundant, this providing the aquatic medium for the vector proliferation. With the onset of summer, irrigation in the paddy fields is usually intensified. The paddy fields and the irrigation channels had been identified as the source of the mosquitoes. Similar seasonal transmission patterns with two peaks had been reported in neighbouring Indian states on Assam and West Bengal [16-18].

**Figure 4:**
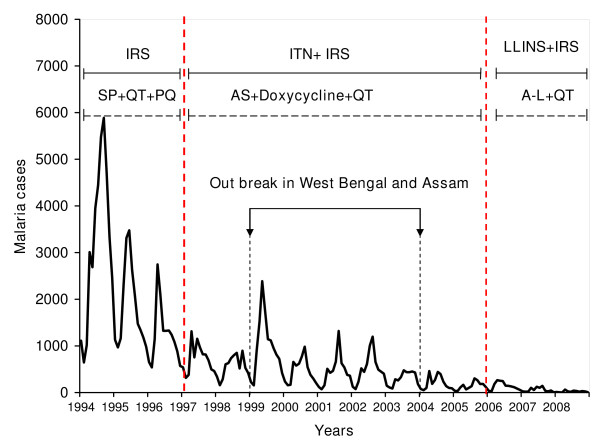
**Malaria trends of the endemic districts of Bhutan with control and treatment measures**. (Note: SP: sulphadoxine-pyrimethamine; QT: quinine; PQ: primaquine; AS:Artesunate; A-L: artemether and lumefantrine).

**Figure 5:**
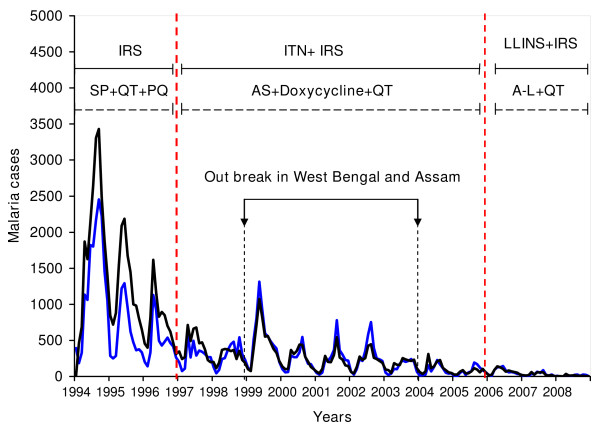
**Malaria trends of the endemic districts of Bhutan with the treatment and control measures**. The black line shows the trend of *Plasmodium falciparum *and blue line shows the trend of *Plasmodium vivax*. (Note: SP: sulphadoxine-pyrimethamine; QT:quinine; PQ: primaquine; AS:Artesunate; A-L: artemether and lumefantrine).

The decrease in the trend of overall districts and most of the districts could be due to a number of factors. One round of IRS with deltamethrin (synthetic pyrethoid) was the main prevention and control measures from 1994 to 1997. The chemicals used for IRS till 1994 was DDT. The introduction of ITN with IRS from 1998 led to significant decrease in the malaria cases. The bed nets were treated every six months and one round of IRS was carried out. In 2006, VDCP introduced LLINs, and distributed over 100,000 LLINs with the grants from GFATM to the seven endemic districts of Bhutan. Similarly, the treatment of *P. falciparum *changed from sulphadoxine and pyrimethamine in 1997 to artesunate and doxycycline between 1997 and 2006. Finally, in 2006 the combination therapy of artemether and lumefantrine (Coartem^®^) was introduced. However, *P. vivax *is still treated with three days regimen of chloroquine and fourteen days primaquine, with quinine as the secondary drug for the treatment of complicated malaria till now (Figure 4). A study in Thailand by Zhou reported sharp drop in falciparum cases when the drug for the treatment of *P. falciparum *was introduced [19]. However, this was not true in case of Bhutan since both types of infection, *P. falciparum *and *P. vivax *infection, decreased proportionately (Figure 5). Therefore, it was concluded that the decrease in the trend could be attributed to the successful implementation of the control measures.

The malaria trend decreased gradually with upsurge again in 1999 till 2003. This was closely related to the increased malaria cases in Assam and West Bengal [16-18]. Bhutan shares eastern half of borders with Assam, three districts that shares the borders are Samdrup Jongkhar, Pemagatshel and Sarpang. While western districts bordering West Bengal are Samtse, Chukha and Dagana. The malaria cases from Samdrup Jongkhar and Sarpang increased during this period. However, no significant increase in malaria cases in the districts bordering West Bengal was noted, despite open border between these districts and West Bengal, India.

The only district to have an increase in trend was Pemagatshel district (Table 4). Three endemic sub-districts of Dechenling, Norbugang and Chorkroling were added to Pemagatshel from Samdrup Jongkhar district in 2006. This could have lead to the increased malaria cases reported from Pemagatshel district. Other plausible reason is the migration of the people for job opportunity since the construction of one of the largest cement plant in Bhutan is under way. This job opportunity could lead to the migration of people both internal as well as expatriates. The risk of increased malaria cases should be anticipated in the coming years.

### Malaria clusters

The identification of clusters in the sub-districts would provide important information on the malaria transmission especially with very few cases as in Bhutan. It had been reported in other studies that malaria transmission usually does not seems to take place at an altitude above 2000 meters [20,21]. However in this study, malaria clusters were persistently observed in sub-districts which are located at an altitude above 2000 meters, like Serthi and Shingkhar Lauri under Samdrup Jongkhar district for the month of May and June over the study period (Figure 2). Malaria clusters in these high altitude districts could be as a result of imported cases from other endemic areas, because most of the businesses and administrations are located in sub-districts, where malaria transmission takes place. People could possibly get the infection from the sub-districts with malaria transmission while they travel for work opportunities and businesses, then manifest clinical signs and symptoms on returning to their villages. This finding could be important to the programme in mitigating the malaria transmission among the internal migrants.

In this study, malaria clusters occurred throughout the year. A number of plausible reasons were hypothesized; firstly, these areas are located in the environmentally favourable region with sub-tropical climate, this facilitates in vector multiplication. Secondly, most of these sub-districts have large areas as the paddy field/wet lands. These paddy fields and irrigation channels could provide breeding sites for the mosquitoes. The main vectors in Bhutan are *Anopheles pseudowillmori *(33%), *Anopheles vagus *(31.5%) and *Anopheles peditaeniatus *(10.3%) [1]. Similar finding of *Anopheles maculates *multiplying in the paddy field had been report by Kankaew [22]. The introduction of community fish farming in these districts in recent years could also be one of the factors for identification of malaria clusters. Common fish that are reared in these fisheries are *Cirrhina cirrhosus *(Mirgal), *Ctenopharyngodon idella *(Grass carp), *Hypopthalmicthys molitrix *(Silver carp), *Cyprinus carpio *(Common carp), *Aristichthys nobilis *(Big head carp), *Catla catla *(Catla), and *Labeo rohita *(Rohu) (personal communications). These fish farming provided aquatic environment for the development of mosquitoes. Fourthly, the introduction of non-immune population through migrations for various purposes could be one of the reasons. Cross-border malaria could be other reasons since borders are quite open and people of either Bhutan or India can easily cross the border. Malaria transmission on either side of border would have a direct effect on malaria cases on either side of border, since malaria transmission is spatially related. As seen in the malaria cluster map, clusters mostly occurred in the sub-districts that share the border with India (Figure 2). Two states that share the border with Bhutan in the east and west are Assam and West Bengal, respectively. Dagana, Chukha and Samtse share border with West Bengal, while Sarpang, Zhemgang, Pemagatshel and Samdrup Jongkhar share border with Assam. These districts particularly Sarpang and Samdrup Jongkhar showed malaria clusters consistently throughout the year. Assam is malaria endemic district and contributes more than 5% of total malaria in India [16]. West Bengal is also considered as malaria endemic state [23], but only some of the sub-districts of Dagana showed clusters while other sub-districts of Chukha and Samtse districts did not have malaria clusters for some unknown reasons.

## Conclusions

There is gradual decrease in the malaria trend in Bhutan with very low malaria incidence. The decrease in the trend could be attributed to the success of control and preventive measures. Malaria clusters vary amongst the districts of the endemic areas. The finding of malaria clusters in the areas where malaria transmissions usually do not occur suggests that the control measures in these regions need to be addressed. The elimination of malaria in Bhutan could be possible with the control measures reinforced in the areas of malaria clusters at the sub-districts.

## Competing interests

The authors declare that they have no competing interests.

## Authors' contributions

KW and JK conceived the study. KW undertook data extraction, statistical analysis, interpretation of results and drafted the manuscript. JK assisted in statistical analysis, interpretation of results and was involved in critical revision of manuscript. PS, TS and SL assisted in statistical analysis, interpretation of results and were involved in the critical revision of the manuscript. NJW assisted in the interpretation of results and revision of the manuscript. All authors read and approved the final manuscript.
